# Peer-teaching: an important skill for all medical students and doctors?

**DOI:** 10.1007/s40037-015-0157-0

**Published:** 2015-01-21

**Authors:** David S. Blanchard

**Affiliations:** School of Medicine, Keele University, ST5 5BG Keele, Staffordshire UK

Research findings from Canada, published in this issue [1], describe the teaching experiences of a small number of postgraduate doctors training in primary care. A first-year resident is observed by a more senior, second-year colleague who provides teaching and feedback on doctor-patient interactions in an ambulatory clinical setting. The interaction between the first- and second-year residents is observed by a faculty preceptor. The doctor undertaking the teaching receives feedback from his junior colleague and from the experienced educator.

It is interesting to note at the outset that all the three levels of clinical experience involved in these processes describe the interactions in extremely positive ways. The first-year resident is provided with a worthwhile educational experience by a colleague he can very easily relate to on an educational, professional and perhaps even social level. The second-year resident not only develops confidence in his capacity to teach but also increases his depth of self-reflection in the clinical setting, an essential skill for doctors at any level of experience. And finally, the faculty preceptor found that ‘the experience of observing and providing feedback of supervision skills promoted reflection of their own teaching methods and effectiveness’. Involvement in these peer-teaching activities had the power to improve professional competencies at three entirely different levels of medical training and practice.

Is this unusual? Perhaps not. There is an increasing amount of published work that has demonstrated the profound benefits of peer-teaching to teachers and learners alike [2–5]. Peer-teaching can provide learners with an effective educational experience from someone they see as being congruent and perceptive of their educational needs; someone with a better understanding of what they do or don’t know: the well-established features of so-called cognitive congruence [6]. Furthermore, peer-teachers not only develop the skills of teaching that are vital for all doctors but also, thereby, increase their own clinical knowledge and other professional skills [7, 8].

Such research and developments have helped to promote an enthusiasm for peer-teaching [5–7] which is surely now to be welcomed and promoted. As described in this issue [1], and elsewhere [9], involvement in peer-teaching can have positive educational outcomes at different levels of medical training and should probably, therefore, be actively advocated for both undergraduate and postgraduate medical training.

Perhaps surprisingly, this recent study [1] found that the doctors involved in the teaching activity felt the need to be monitored by a faculty preceptor to ‘ensure patient safety and adequacy of the educational experience’ and, quite rightly, the authors indicate the resource implications of requiring supervision for all such peer-teaching interactions. This perceived need for adequate supervision might be due to the complexity of the task involved in this specific situation: teaching a peer and, at the same time, being responsible for patient care. This contrasts with most other published medical peer-teaching experiences, especially at an undergraduate level, which usually consist of a specific teacher-learner interaction without, understandably, direct responsibility for patient care [7].

This creates the image of a peer-teaching ladder of progression where initial peer-teaching interactions are less complex, involving a teacher-learner relationship based perhaps in a non-clinical environment; an example might be a third-year medical student teaching anatomy to first-year students in a classroom setting. Climbing up the peer-teaching ladder might involve the teaching of skills in a clinical environment but without the responsibility for patient clinical care; for example, fifth-year students providing bedside teaching to third-year students in a hospital setting. Further progression might lead to the situation described in the study by Ince-Cushman et al. [1], with the teacher having both teaching responsibilities to the learner and, at the same time, being clinically responsible for the patient involved in the teaching; very much our daily experience as GP tutors teaching medical students in primary care. Such a peer-teaching ladder of progression could provide a meaningful model of medical peer-teacher development to help to reassure, develop and guide aspiring undergraduate and postgraduate peer-teachers.

So it is possible that the resident doctors investigated by Ince-Cushman et al. [1] lacked confidence in their own teaching abilities, and felt reassured by the presence of a more experienced doctor and educator, simply because they had missed out some of the lower rungs of this peer-teaching ladder and found themselves in a quite complex teaching situation of which they had no previous experience. We are not told, but this might have been their very first exposure to peer-teaching and so understandably they felt uncertain and unsure of their roles and abilities. Add to that the requirement for safe and effective patient care and their lack of confidence would seem quite understandable and appropriate. Further monitoring of these same doctors, were they to continue to be involved in peer-teaching, might demonstrate a growth of skills and confidence leading to a reduced need for supervision.

Yet would these findings have been the same if the registrars involved had already started to climb the peer-teaching ladder? What if they had previously experienced and taken part in peer-teaching activities throughout their undergraduate courses? Would they still feel the need for continued supervision if they had been taught how to teach, and had been provided with opportunities to teach, whilst they were medical students? Increasingly, peer-teaching experiences are provided at an undergraduate level in medical schools [7], and some courses, including our own at Keele, even provide students with the opportunity to learn some of the basic skills of medical education and teaching [10]. Such initiatives should certainly lead to the development of young doctors with a greater confidence in their own abilities to teach.

The trainee doctors involved in peer-teaching in the Canadian study [1] were defined as ‘in good academic standing’. But opportunities to learn about medical education and to teach should not be restricted to the more enthusiastic or higher achieving students and doctors. All doctors need to be effective teachers of patients, colleagues, politicians, society and the global community: the skills required should be taught to all learners throughout all undergraduate and postgraduate medical curricula.

There are, nevertheless, still many unanswered questions in the educational field of medical peer-teaching. For example, will involvement in peer-teaching lead to a better development of professional values and behaviour as has been suggested [8]? How does learning to teach and peer-teaching affect subsequent abilities as a qualified doctor? Can participation in peer-teaching at undergraduate and postgraduate levels lead to enhanced skills in patient education and communication? Will attendance on an undergraduate teaching course promote interest and involvement in a career in academic medicine or medical education? Some of us involved in peer-teaching believe that it leads to better student teachers, an enthusiasm for medical education and more highly trained and effective doctors. Such assumptions, however, remain to be fully researched and confirmed.
